# Methylation of LINE-1 Retroelement in People with Type 1 Diabetes

**DOI:** 10.3390/genes16070759

**Published:** 2025-06-28

**Authors:** Andromachi Katsanou, Charilaos Kostoulas, Evangelos Liberopoulos, Agathocles Tsatsoulis, Ioannis Georgiou, Stelios Tigas

**Affiliations:** 1 Department of Endocrinology, University of Ioannina, 45110 Ioannina, Greece; 2 Department of Internal Medicine, Hatzikosta General Hospital, 45445 Ioannina, Greece; 3 Laboratory of Medical Genetics, Faculty of Medicine, School of Health Sciences, University of Ioannina, 45110 Ioannina, Greece; 4 First Department of Propaedeutic Internal Medicine, Medical School, National and Kapodistrian University of Athens, Laiko General Hospital, 11527 Athens, Greece

**Keywords:** DNA methylation, LINE-1 retroelement, type 1 diabetes

## Abstract

Introduction: Emerging research indicates that alterations in the methylation of retrotransposons may contribute to genomic instability and cellular aging in various autoimmune disorders and diabetes mellitus (DM). As relevant information for people with type 1 diabetes mellitus (PwT1D) is limited, we aimed to investigate long interspersed nuclear element-1 (LINE-1) methylation status in this population. Methods: DNA methylation levels and patterns of LINE-1 were examined in the peripheral blood of 35 PwT1D and 28 healthy controls (age- and sex-matched), by using the COmbined Bisulfite Restriction Analysis methodology (COBRA). Results: Total LINE-1 methylation rate (mC) was higher in PwT1D compared to controls [47.3% (46.6–47.8%) vs. 46.5% (44.7–47.3%), *p* < 0.05]. The partial LINE-1 methylation pattern (uCmC) was less frequently observed in patients vs. controls [28.4% (24.7–33.3%) vs. 33.1% (27.8–37.9%), *p* < 0.05]. Prevalence of other methylation patterns [partially methylated (mCuC), hypermethylated (mCmC) and hypomethylated (uCuC)] was similar in the two groups. Furthermore, levels of fasting glucose and glycated hemoglobin (HbA1c) were positively associated with total methylation (mC) [Spearman’s rho = 0.380, *p* = 0.002 and rho = 0.342, *p* = 0.006, respectively], but negatively associated with the partially methylated (uCmC) pattern [Spearman’s rho = −0.383, *p* = 0.002 and rho = −0.270, *p* = 0.033, respectively]. The LINE-1 (uCmC) methylation pattern was negatively associated with the age at diagnosis of T1D [Spearman’s rho = −0.341, *p* = 0.049], but positively associated with disease duration [Spearman’s rho = 0.388, *p* = 0.021]. Conclusions: PwT1D were found to have higher total LINE-1 methylation rate (mC) compared to healthy controls. The partial methylation pattern (uCmC) was less frequently observed in these patients and was negatively associated with the glycemic status and the age at diagnosis of T1D, while demonstrating a positive correlation with disease duration.

## 1. Introduction

Type 1 diabetes (T1D) is marked by the immune-mediated destruction of pancreatic islet cells, typically preceded by the appearance of islet-specific autoantibodies. Its development is influenced by a complex interplay of genetic predisposition and environmental triggers; several major histocompatibility complex (MHC) and non-MHC loci have been identified [1,2,3], but also epigenetic factors seem to play a role [4,5]. Recent evidence demonstrated the connection between epigenetic mechanisms (such as DNA methylation) and hyperglycemia or late complications in PwT1D [6]. DNA methylation changes may partly explain the increasing incidence of diabetes over the past decades [7,8,9]. We have recently observed similar total Alu methylation rates but differences in partial Alu methylation patterns in patients with T1D compared to healthy controls [10,11].

LINE-1 and Alu elements are the most abundant retrotransposons, comprising approximately 40% of the human genome [12,13]. There are over 500,000 copies of LINE-1 that are able to replicate, integrate themselves in new positions throughout the human genome and to move autonomously by encoding the enzymatic mechanism necessary for their transposition [14,15]. LINE-1 retroelement exceeds 6000 nucleotides in length (nt) and exists in over 100,000 copies, constituting 18% of the genome; however, only 100 of these are potentially active [15,16]. Functional LINE-1 elements are estimated to be 6–7 kb long and generally possess a long 5’ end with RNA polymerase II promoter activity, along with coding open reading frames ORF1 and ORF2 [17,18].

LINE-1 elements have been implicated in disease pathogenesis through various mechanisms, including insertional mutagenesis, disruption of chromatin architecture, and homology-driven deletions that can modulate nearby gene expression [19]. Despite these associations, the precise triggers and timing of retrotransposon activation remain largely unclear [15]. Notably, retrotransposons account for nearly one-third of the genome’s methylation landscape [20]. LINE-1 hypomethylation has been recognized as a hallmark of aging [13,19] and is frequently used as a proxy for assessing global DNA methylation levels [21,22,23,24,25]. Advances in high-resolution genomic tools—such as single-cell RNA sequencing (scRNA-seq), RNA in situ conformation sequencing (RIC-seq), and chromosome conformation capture techniques (Hi-C)—have substantially enhanced our understanding of how retrotransposons influence genomic regulation and evolutionary dynamics [26]. Emerging evidence suggests that changes in the methylation of retroelements, including LINE-1, contribute to the global hypomethylation and genomic instability observed in various cancers, autoimmune conditions, and diabetes mellitus [15,20,27,28]. While numerous studies have linked LINE-1 methylation to metabolic disturbances such as insulin resistance, hyperglycemia, obesity, and cardiovascular risk, these findings are often inconsistent and predominantly pertain to type 2 diabetes (T2D) [11]. To our knowledge, there is currently a lack of data on LINE-1 methylation patterns in individuals with type 1 diabetes (T1D).

The present study aimed to examine (i) the total methylation and the different methylation patterns of LINE-1 element in PwT1D compared to healthy controls and (ii) whether methylation differences correlate with glycemic control in this patient population.

## 2. Materials and Methods

### 2.1. Study Design and Participants

In this case-control study we used the combined bisulfite restriction analysis (COBRA) method to assess the total DNA methylation and methylation patterns of LINE-1 element in the peripheral blood of PwT1D and healthy controls, matched for age and sex. Study participants comprised of 35 PwT1D, who visited the Department of Endocrinology and Diabetes outpatient clinic, at the University Hospital of Ioannina, and 28 healthy individuals matched for age and sex who attended the hospital outpatient clinic for a routine health examination. Inclusion criteria were (a) diagnosis of type 1 diabetes according to current criteria, (b) disease duration of at least 1 year and (c) age between 18 and 70 years. Exclusion criteria included smoking [29], history of autoimmune disease or cancer [30], poorly controlled hypertension (>140/90 mmHg) and overweight or obesity (body mass index, BMI > 25 Kg/m^2^) [20,31,32,33,34]. According to previous scientific data, the aforementioned factors may potentially influence the methylation status of retroelements [34,35,36]. The study was approved by the Ethics Committee of Ioannina University Hospital (approval number #1586) and all participants provided informed consent prior to enrollment, in accordance with the Helsinki Declaration.

### 2.2. Laboratory Methods

The COBRA-interspersed repetitive sequence PCR method was used. This is a very reliable semi-quantitative method for assessing DNA methylation. It enables the detection of methylation at multiple CpG sites, including cases where pyrosequencing fails to reveal a clear methylation pattern [37].

### 2.3. DNA Extraction and Bisulfate DNA Modification

DNA was extracted from circulating cells in peripheral blood by using DNA Blood Mini kit (Qiagen, Hilden, German) according to the manufacturer’s guideline. A Quawell Q500 spectrophotometer was used to measure DNA concentration and for the conversion of 1mg of the extracted DNA we use a bisulfite EpiTech methylation kit (Qiagen, German), according to the manufacturer’s protocol. To assess methylation levels of LINE-1, the sodium-bisulfite-treated DNA was amplified in each sample by PCR, using 0.2 mM of deoxynucleotide triphosphate, 1 U of HotStarTaq DNA Polymerase (Qiagen, Germany), 1 mM of magnesium chloride, and 0.3 μM primer pairs (LINE-1-F: 5′-CCGTAAGGGGTTAGGGAGTTTTT-3′ and LINE-1-R: 5′-RTAAAACCCTCCRAACCAAATATAAA-3′).

**Figure 1:**
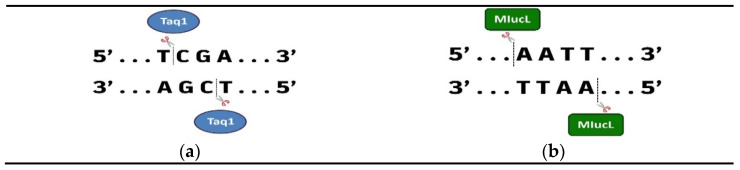
(**a**) The TaqI enzyme specifically identifies methylated cytosines in CpGs dinucleotides and cuts them at the indicated points. (**b**) The MIuCI enzyme cuts at unmethylated regions when it specifically identifies the sequence.

Bisearch analysis revealed that the primers will amplify about 3500 PCR fragments, with representative LINE-1s that are scattered throughout the genome. For LINE-1 amplification, the following PCR conditions were used: 95 °C for 15 min, 35 cycles of 95 °C for 45 s, 53 °C for 30 s, 72 °C for 45 s and a final extension at 72 °C for 7 min. Then, after PCR amplification, the LINE-1 PCR products (160 bp) were digested using firstly 4U of MluCI enzyme (New England Biolabs, Ipswich, MA, USA) and then 2U of TaqI enzyme (New England Biolabs, Ipswich, MA, USA) in CutSmart buffer (New England Biolabs, Ipswich, MA, USA) and incubated at 65 °C overnight (Figure 1). The digested PCR products were finally analyzed by 8% acrylamide gel and SYBR green nucleic acid gel stain (Gelstar, Lonza, Rockland, ME, USA) (Lonza, Biologics Inc. Houston, TX, USA). The intensity of the LINE-1 methylation band was measured and observed by phosphorimager, using 1D image analysis software (kodaks v3.6.4, KODAK, Rochester, NY, USA).

### 2.4. Methylation Analysis

The COBRA-LINE-1 assay yielded four distinct methylation patterns based on the status of two CpG dinucleotides studied: (1) an unmethylated pattern (uCuC), indicating hypomethylation at both CpGs, two partially methylated patterns (2) (mCuC) and (3) (uCmC) and finally (4) a methylated pattern (mCmC), indicating hypermethylation at both CpGs.

**Figure 2:**
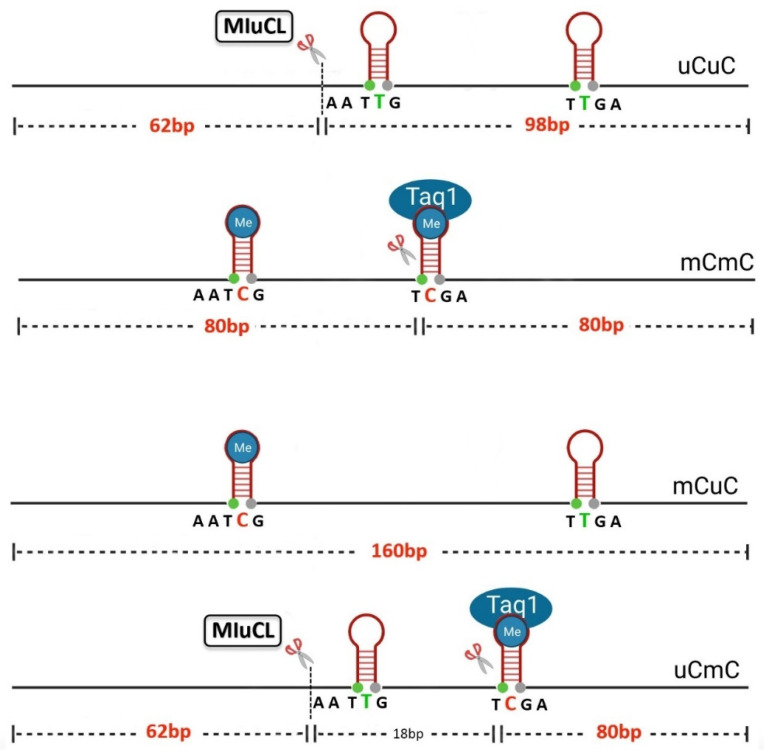
The methylation pattern of LINE-1 is derived from the methylation status of two CpG sites specifically identified by the enzymes MIuCl and TaqI: In the methylated LINE-1 pattern (mCmC) the two sites are recognized by TaqI which cuts only the second methylated CpG dinucleotide resulting in 2 products of 80 bp. Conversely, in the case of demethylated pattern (uCuC), the enzyme MIuCl cuts only the first unmethylated CpG dinucleotide and creates 2 bands of 62 and 98 bp. Regarding partially methylated LINE-1, there are 2 patterns: in (uCmC) the unmethylated CpG dinucleotide of size 62 bp is identified by MIuCl, while the methylated CpG dinucleotide of size 80 bp is recognized by TaqI, resulting in the creation of 3 bands of 18 bp, 62 bp and 80 bp. The 18 bp band is not included in the study. In (mCuC), the methylated CpG dinucleotide of size 62 bp and the unmethylated CpG dinucleotide of size 80 bp are not recognized by the restriction enzymes MIuCl and TaqI and in polyacrylamide electrophoresis will show a band of 160 bp. The blue circles represent methylated cytosine and the hollow ones represent unmethylated cytosine (converted to thymine).

To distinguish these patterns, the PCR products were digested with the restriction enzymes TaqI and MluCI, generating DNA fragments of 160, 98, 80, and 62 bp, as shown in Figure 2. The LINE-1 methylation level of each pattern was calculated to obtain the exact percentage, as follows: Each band’s intensity was divided by the length (bp) of the double-stranded DNA: %160 bp/160 = A, %98/94 = B, %80/78 = C and %62/62 = D. Then, the LINE-1 methylation frequency in each pattern was calculated as follows:

- Total methylated loci % mC = 100 × (C + A)/(C + 2A + B + D);
- Methylated pattern % mCmC = 100 × ((C − D + B)/2)/((C − D + B)/2 +D + A);
- Unmethylated pattern % uCuC = 100 × B/((C − D + B)/2 +A + D);
- Partially methylated pattern % uCmC = 100 × ((D − B)/((C − D + B)/2) +A + D);
- Partially methylated pattern % mCuC = 100 × A/((C − D + B)/2 +A + D);

In every experiment, DNA samples isolated from Jurkat (Leukemia), Daudi (Leukemia) and HeLa (cervical cancer) cell lines were used as positive controls to minimize inter-assay variability of the COBRA method [38]. DNA extraction and methylation analysis were conducted at the Laboratory of Medical Genetics, University of Ioannina.

The total LINE-1 methylation rate (mC), as well as the prevalence of different LINE-1 methylation patterns of the two studied CpG dinucleotides were compared between cases and controls. The different methylation patterns comprised of: (a) Hypermethylated pattern (mCmC), (b) hypomethylated pattern (uCuC), (c) partially methylated pattern (mCuC) and (d) partially methylated pattern (uCmC). In addition, we analyzed total methylation and methylation patterns according to sex as well as age at diagnosis.

### 2.5. Statistical Analysis

The Shapiro–Wilk test was employed to assess the normality of continuous variables. Variables not following a normal distribution were summarized using medians and interquartile ranges (IQRs), while categorical variables were reported as percentages. Group comparisons between cases and controls for baseline characteristics and LINE-1 methylation patterns were performed using the Mann–Whitney U test for non-normally distributed continuous variables and the Chi-squared test for categorical data, as appropriate. 

Associations between LINE-1 methylation levels, specific methylation patterns, and patient characteristics were further evaluated using Spearman’s rank correlation coefficient. Based on limited published literature and our preliminary data in patients with type 1 diabetes we estimated that a sample size of at least 25 patients in each group would provide a power of approximately 80% to detect a difference of 1% in LINE-1 methylation between the two groups (α = 0.05, estimated SD of up to 1.8). The SPSS version 25 by DatAnalysis was used for statistical analyses. A *p*-value of 0.05 was considered as the threshold for statistical significance.

## 3. Results

Characteristics of study participants and LINE-1 methylation patterns are depicted in Table 1. As expected, PwT1D had higher fasting glucose [113 (94.0–146.0) vs. 79.5 (75.5–89.0,) *p* < 0.05], and HbA1c levels [7.3% (6.8–7.9) vs. 4.9% (4.7–5.2) *p* < 0.05] compared with controls. Participants’ median age at diagnosis was 14 years (9.0–21.0), corresponding to a median of 12 (4.0–20.5) years of T1D duration at the time of study enrollment.

### 3.1. Total Methylation and Methylation Patterns of LINE-1 Element in Patients with T1D Compared to Healthy Controls

**Figure 3:**
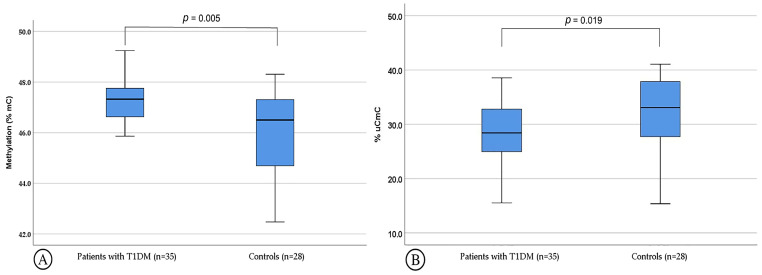
Percentage of (**A**) total methylatation (mC) and (**B**) partially methylated LINE-1 pattern (uCmC) in patients with T1D and healthy controls. Results are presented as box plots: the boxes represent the interquartile ranges (25th to 75th percentile), the median lines represent the 50th percentile and the whiskers represent the minimum and maximum values.

A statistically significant difference between PwT1D and age in sex-matched controls was identified in the total (mC); specifically, a higher percentage of (mC) was observed in PwT1D [47.3% (46.6–47.8%) vs. 46.5% (44.7–47.3%), *p* = 0.005]. In addition, the partial LINE-1 methylation pattern (uCmC) was less frequently observed in T1D patients compared to controls [28.4% (24.7–33.3%) vs. 33.1% (27.8–37.9%), *p* = 0.019] (Figure 3). Finally, the prevalence of partial methylation pattern (mCuC) and the hypermethylated (mCmC) and hypomethylated (uCuC) patterns were similar in PwT1D and controls (Table 1).

### 3.2. Association of LINE-1 Total Methylation and Methylation Patterns with Patients’ Characteristics and Glycemic Control 

In correlation analyses, it was observed that both fasting glucose and HbA1c were positively associated with total (mC) [Spearman’s rho = 0.380, *p* = 0.002 and rho = 0.342, *p* = 0.006 respectively]. On the contrary, both fasting glucose and HbA1c were negatively associated with the partially methylated (uCmC) pattern [Spearman’s rho = −0.383, *p* = 0.002 and rho = −0.270, *p* = 0.033, respectively]. Νo associations between fasting glucose or HbA1c and the other LINE-1 methylation patterns [(mCmC), (mCuC), (uCuC)] were detected (Table 2).

Moreover, no other association of total (mC) or all the different LINE-1 methylation patterns (mCmC), (uCuC), (mCuC) and (uCmC) with age, sex or the presence of chronic diabetes complications was detected (Table 2).

### 3.3. Association of LINE-1 Total Methylation and Methylation Patterns with the Age at Diagnosis of T1D and the Duration of Diabetes 

No association was observed between (mC) and age at diagnosis of T1D or duration of diabetes. Similarly, no correlation was observed between these parameters and the LINE-1 methylation patterns [(uCuC), (mCuC) and (mCmC)], except for (uCmC) methylation pattern (Table 2). Specifically, the (uCmC) LINE-1 pattern was negatively associated with the age at diagnosis of T1D [Spearman’s rho = −0.341, *p* = 0.049], and positively associated with the duration of the disease [Spearman’s rho = 0.388, *p* = 0.021] (Table 2).

Finally, the association between total methylation and the various methylation patterns of LINE-1 element was cross-checked in both groups (patients and controls). In the PwT1D group a negative correlation was observed between (mC) and (uCmC) [rho = −0.364, *p* < 0.05], whilst in the control group, (uCmC) was not associated with (mC). In addition, this partially methylated pattern (uCmC) was negatively associated in both groups (patients and controls) with totally methylated pattern (mCmC) [rho = −0.529, *p* = 0.004 and rho = −0.342, *p* < 0.05., respectively], but no association was found with the unmethylated pattern (uCuC).

## 4. Discussion

The present study assessed the LINE-1 total methylation (mC) and its various methylation patterns in PwT1D compared to age- and sex-matched healthy controls. PwT1D were found to have higher LINE-1 total mehtylation (mC), but lower levels of the partial LINE-1 (uCmC) methylation pattern. In correlation analyses, a positive association between (mC) and glycemic status (fasting glucose or HbA1c), but a negative association between (uCmC) pattern and fasting glucose or HbA1c was observed. Furthermore, this pattern was negatively associated with the age at diagnosis of T1D, and positively with the duration of T1D at the time of enrollment into the study. Moreover, a negative association between (mC) and (uCmC) pattern was observed in PwT1D but not in the control group, suggesting that this alteration in methylation status was specific for patients with T1D. Finally, no association of total (mC) or all the different LINE-1 methylation patterns with age, sex or the presence of chronic complications of diabetes was detected.

**(i)**  LINE-1 methylation and T1D

Scientific evidence indicates that both genetic and environmental factors influence T1D genetic loci and are mediated by differential levels or patterns of DNA methylation (hyper- or hypomethylation) [39,40,41]. Interestingly, certain T1D associated genes, such as *HLA-DQB1* and *GAD2*, were found to be either hypermethylated or hypomethylated [5]. Moreover, other authors tried to summarize the major HLA loci related to T1D (HLA region 6p21.32) for LINE-1 and Alu subfamilies (DQA1, DQB1, DRB1), annotated by RepeatMasker (a program searching DNA sequences for interspersed repeats) [15,42]. Also, prolonged hyperglycemia led to aberrant epigenetic marks that persisted even after the restoration and maintenance of euglycemia, suggesting the epigenetic processes may affect the process of “metabolic memory” [43,44,45,46,47]. Increased levels of peripheral blood unmethylated preproinsulin DNA have been observed in persons with newly diagnosed T1D compared to healthy controls [48]. Notably, a recent epigenome-wide association study (EWAS) identified inverse associations between DNA methylation at cg19693031 (chromosome 1, Thioredoxin-Interacting Protein [TXNIP]) and cg21534330 (chromosome 17, Casein Kinase 1 Isoform Delta) with HbA1c levels, indicating that lower TXNIP methylation may be linked to poorer glycemic control in T1D [7]. Findings from the Diabetes Control and Complications Trial and its follow-up, the Epidemiology of Diabetes Interventions and Complications (EDIC) study, further suggest that methylation at specific CpG sites may play a role in metabolic memory and the association between glycemic status and late complications in diabetes [44].

**(ii)**  Correlation between LINE-1 methylation and hyperglycemia

In the present study, a positive association between glycemic status (fasting glucose or HbA1c) and total methylation (mC) of LINE-1 retroelement was observed, whereas the association between (uCmC) methylation pattern and fasting glucose or HbA1c was negative. This methylation pattern was also negatively associated with the age at diagnosis of T1D and positively with the duration of the disease.

Studies examining LINE-1 methylation and hyperglycemia have produced various findings. Some studies report a positive association between LINE-1 methylation and hyperglycemia or diabetes [21,49], while others found an inverse relationship with fasting glucose and metabolic syndrome (MS) [31,50,51,52,53]. Still, a few studies report no significant association [54,55]. For example, Pearce et al. [49] linked higher LINE-1 methylation with elevated fasting glucose, cholesterol, triglycerides, and LDL, suggesting a potential role for LINE-1 as an epigenetic biomarker of metabolic risk. Conversely, a prospective study showed that lower baseline LINE-1 methylation predicted increased risk of T2D, impaired fasting glucose (IFG), and impaired glucose tolerance (IGT), independent of traditional risk factors [51]. Similarly, in a 14-year cohort of T2D patients, higher LINE-1 methylation was inversely associated with diastolic blood pressure, cholesterol/HDL ratio and eGFR, but not with HbA1c [31]. Additional studies have produced mixed results. For example, reduced LINE-1 methylation was linked with diabetes, aging, and coronary heart disease (CHD) [51], and negatively associated with fasting glucose in visceral fat tissue [53]. On the contrary, Carraro et al. found a positive correlation between LINE-1 hypermethylation and insulin resistance, adiposity (BMI, WC) and poor diet quality [50].

A review of 20 observational and interventional studies concluded that LINE-1 methylation is related to obesity-related disorders, insulin resistance, T2D, and cardiovascular disease (CVD) [20]. Nonetheless, the overall evidence remains contradictory, as both positive and negative associations have been reported [56,57,58,59].

**(iii)**  Alteration of total LINE-1 methylation and methylation patterns in T1D

In the present study, total methylation (mC) of the LINE-1 element was higher, and the partial methylation pattern (uCmC) was less frequent in PwT1D compared to age- and sex-matched healthy controls; no differences were observed in the other methylation patterns between the two groups.

In previous studies, total DNA methylation was found to be elevated in monozygotic twins with T1D [4]. Fradin et al. reported variations in methylation between PwT1D and healthy controls identifying a specific 3-CpG-hypomethylation pattern unique to these patients, although this CpG methylation pattern did not appear to correlate with levels of HbA1c or disease duration [41]. On the other hand, no variations in methylation patterns in umbilical cord blood were observed in children who progressed to T1D versus those who remained healthy [8].

Although recent knowledge about alterations in total methylation or different methylation patterns of LINE-1 in T1D is limited, previous studies have explored this issue in patient populations with other autoimmune diseases. For example, methylation levels of LINE-1 in the neutrophils from patients with systemic lupus erythematosus were significantly lower compared to healthy controls and this hypomethylation did not correlate with disease activity. Interestingly, the percentage of the (mCuC) pattern of LINE-1 in neutrophils was higher in the patients compared to controls, whilst no significant difference was observed in the (uCmC) pattern between the two groups [30].

In patients with psoriasis vulgaris, LINE-1 hypomethylation and an increase in (uCuC) methylation pattern was found (using the COBRA-LINE-1 method) in the keratinocytes of patients when compared with healthy controls. Moreover, (uCmC) mehtylation pattern was significantly lower in patients with severe psoriasis compared to those with mild disease. Yooyongsatit et al. suggested that modification in LINE-1 methylation may affect the gene expression resulting in a phenotypic change of the psoriatic skin and that (uCuC) and (uCmC) LINE-1 methylation patterns may be used as biomarkers for psoriasis [60].

**(iv)**  Correlation of LINE-1 methylation with age or sex

In our study population, no association of total LINE-1 methylation (mC) or any of the LINE-1 methylation patterns with age or gender was detected.

Limited information is available about the role of retroelements such as LINE-1, in T1D during aging. The identification of methylation changes that occur before the diagnosis of clinical diabetes or even prior to the onset of islet autoimmunity implies a potential role for epigenetics in the pathogenesis of the disease [9,61,62,63,64]. Although recent studies have linked the derepression of LINE-1 elements with aging and age-related diseases [65,66], other studies failed to observe any such association [51].

Our observations are consistent with those of previous studies reporting that age and sex do not influence LINE-1 methylation [29]. Previous studies in patients with T2D found no association between global LINE-1 methylation and sex when compared to healthy controls [31]. Likewise, Wu et al. found no differences in DNA methylation of LINE-1 in the cross-talking of leukocyte telomere length (LTL) [21]. However, in other studies, levels of LINE-1 DNA methylation in peripheral blood of patients with T2D were higher in males compared to females [20].

**(v)**  Strengths and Limitations

The present study is the first to report on differences in LINE-1 element methylation in PwT1D compared with age- and sex-matched controls. We used COBRA-interspersed repetitive sequence PCR, a very accurate semiquantitative methylation analysis method, that can detect more than one CpG site. Certain limitations should however be taken into consideration. Firstly, because of the relatively small sample size, it is possible that differences in other methylation patterns between patients with T1D and controls and potential associations of methylation patterns with patient characteristics may have been missed. As the sample size of our study was insufficient to support multivariable modeling, we used nonparametric tests, as previously mentioned. Second, we did not perform analyses for possible confounders, such as physical activity or dietary habits, which are known to potentially affect the human epigenome. Furthermore, there is growing evidence that environmental exposures can modulate DNA methylation patterns at key metabolic and immune loci [67,68,69], highlighting the importance of exploring the interplay between metabolic regulation and epigenetic mechanisms in future studies. Third, due to the lack of data on other comorbidities or concurrent medication in our population of PwT1D, the possible impact of the aforementioned factors in LINE-1 methylation could not be tested. Lastly, taking into account that this is an observational study we cannot infer causality between LINE-1 methylation and glycemic indices (glucose and HbA1c) and thus the possibility that LINE-1 methylation is the consequence and not the cause of the hyperglycemia cannot be excluded.

**(vi)**  Future perspectives

Retrotransposons, such as LINE-1 elements, can influence transcription through various mechanisms, including acting as alternative promoters or enhancers. Quantifying LINE-1 methylation is often used as a surrogate marker for global DNA methylation (DNAm) levels [11,21,22,23,24,25], though its impact can vary depending on its genomic location. For instance, promoter CpG island hypermethylation is typically associated with gene silencing, while hypomethylation often correlates with increased gene expression [70]. Environmental factors may alter the methylation status of LINE-1 elements through gene–environment interactions. However, it remains unclear whether these epigenetic alterations are a cause or consequence of hyperglycemia and disease progression in individuals with DM.

According to our findings, although in PwT1D increased frequency of the partially methylated pattern (uCmC) was associated with worse glycemic indices, younger age at diagnosis and increasing duration of the disease, no correlation with the (mCuC) pattern was detected. This could probably be explained by the fact that hyperglycemia may affect LINE-1 methylation via distinct mechanisms by increasing or decreasing the number of hypo- and hypermethylated loci. Interestingly, a negative association between pattern (uCmC) and (mC) was observed only in PwT1D, but not in the control group. Evidence suggests that measuring overall LINE-1 methylation levels may not be sufficient and that additionally assessing LINE-1 methylation patterns may better reflect LINE-1 changes in various conditions [29]. This is consistent with previous observations reporting that certain methylation patterns are more sensitive in revealing alterations in DNAm and thus they could probably serve as potential novel markers of certain diseases [10,13,35,59,60,71].

Molecular mechanisms causing LINE-1 loss or gain methylation are ambiguous. Therefore, the evaluation of global DNAm offers a rather simplified view of epigenetic imbalance, as it does not recognize, either qualitatively or quantitatively, the co-existence of hypermethylation and hypomethylation within different genes in the same cell [72]. The application of sophisticated methods for DNAm analysis and future research using long-read sequencing technology may provide reliable data and enhance our understanding of the regulation of LINE-1 retrotransposons and their significance for T1D pathogenesis and disease progression.

**(vii)**  Conclusion

A higher total LINE-1 methylation rate (mC), but lower partial methylation pattern (uCmC), was observed in PwT1D compared to age- and sex-matched healthy controls. Moreover, this partial methylation pattern was negatively associated with the glycemic status and the age at diagnosis of T1D, but positively with the duration of the disease. Patterns of DNA methylation, beyond overall methylation levels, may provide valuable insights into epigenetic alterations associated with T1D. However, our findings require validation in larger observational studies that examine a broader range of retroelements. 

## Figures and Tables

**Table 1:** Subject characteristics and LINE-1 methylation patterns in PwT1D and controls.

Variable	Patients with T1D	Controls	*p*-Value
	(n = 35)	(n = 28)	
**Subject Characteristics**			
Age (years, median, IQR)	28.0 (19.0–39.0)	28.0 (20.0–31.5)	0.658
Males/Females (%)	56.7/43.3	56.7/43.3%	0.866
Systolic blood pressure (mmHg, median, IQR)	125.0 (110.0–130.0)	115.0 (100.0–125.0)	0.316
Diastolic blood pressure (mmHg, median, IQR)	75.0 (70.0–85.0)	70.0 (65.0–80.0)	0.751
BMI (kg/m^2^)	23.0 (21.0–24.0)	22.0 (21.0–23.0)	0.548
Fasting glucose (mg/dL, median, IQR)	113 (94.0–146.0)	79.5 (75.5–89.0)	<0.05 *
HbA1c (%,median, IQR)	7.3 (6.8–7.9)	4.9 (4.7–5.2)	<0.05 *
**LINE-1 methylation**			
mC (%, median, IQR)	47.3 (46.6–47.8)	46.5 (44.7–47.3)	0.005 *
mCmC (%, median, IQR)	35.3 (26.0–48.3)	31.8 (24.2–44.0)	0.534
uCmC (%, median, IQR)	28.4 (24.7–33.3)	33.1 (27.8–37.9)	0.019 *
mCuC (%, median, IQR)	16.4 (11.3–25.1)	15.9 (12.3–19.4)	0.463
uCuC (%, median, IQR)	18.8 (12.1–23.4)	18.0 (13.8–24.4)	0.251

Abbreviations: T1D = type 1 diabetes mellitus, IQR = interquartile range. * Correlation is considered significant at the 0.05 level (2-tailed). *p* values in bold text indicate a statistically significant difference.

**Table 2:** Association of the different LINE-1 methylation patterns with patient characteristics.

Variable	mC (%)	mCuC (%)	uCmC (%)	uCuC (%)	mCmC (%)
**Age**	rho = 0.096, *p* = 0.456	rho = −0.109, *p* = 0.396	rho = 0.009, *p* = 0.947	rho = −0.203, *p* = 0.11	rho = 0.156, *p* = 0.223
**Age at diagnosis**	rho = 0.117, *p* = 0.509	rho = 0.091, *p* = 0.608	rho = −0.341 *, *p* < 0.05	rho = 0.070, *p* = 0.696	rho = −0.015, *p* = 0.933
**Sex (female)**	rho = 0.140, *p* = 0.281	rho = −0.202, *p* = 0.189	rho = 0.198, *p* = 0.126	rho = −0.168, *p* = 0.195	rho = 0.129, *p* = 0.322
**Duration of disease**	rho = −0.020, *p* = 0.911	rho = −0.046, *p* = 0.792	**rho = 0.388 *p* < 0.05**	rho = −0.233, *p* = 0.177	rho = 0.026, *p* = 0.0883
**Fasting glucose**	rho = 0.380 *, *p* < 0.05	rho = 0.022, *p* = 0.864	rho = −0.383 *, *p* = 0.002	rho = −0.113, *p* = 0.376	rho = 0.122, *p* = 0.340
**HbA1c**	**rho = 0.342, *p* < 0.05**	rho = 0.087, *p* = 0.496	rho = −0.270 *, *p* = 0.033	rho = −0.111, *p* = 0.385	rho = 0.056, *p* = 0.665

Abbreviations: rho = Spearman’s rank correlation coefficient. * Correlation was considered significant at the 0.05 level (2-tailed). *p*-values in bold text indicate a statistically significant difference.

## Data Availability

Data presented in this study are available upon reasonable request from the corresponding author. The data are not publicly available due to ethical reasons.
